# Diagnosis and Management of the Cryopyrin-Associated Periodic Syndromes (CAPS): What Do We Know Today?

**DOI:** 10.3390/jcm10010128

**Published:** 2021-01-01

**Authors:** Tatjana Welzel, Jasmin B. Kuemmerle-Deschner

**Affiliations:** 1 Pediatric Rheumatology and Autoinflammation Reference Center Tuebingen (arcT), University Children’s Hospital Tuebingen, D-72076 Tuebingen, Germany; tatjana.welzel@ukbb.ch; 2 Pediatric Pharmacology and Pharmacometrics, University Children’s Hospital Basel (UKBB), University of Basel, CH-4031 Basel, Switzerland

**Keywords:** CAPS, FCAS, MWS, CINCA, NOMID, hearing loss, urticarial-like rash, autoinflammatory disease, anti-IL-1 treatment

## Abstract

The cryopyrin-associated periodic syndromes (CAPS) are usually caused by heterozygous *NLRP3* gene variants, resulting in excessive inflammasome activation with subsequent overproduction of interleukin (IL)-1β. The CAPS spectrum includes mild, moderate, and severe phenotypes. The mild phenotype is called familial cold autoinflammatory syndrome (FCAS), the moderate phenotype is also known as Muckle–Wells syndrome (MWS), and the neonatal-onset multisystem inflammatory disease (NOMID)/chronic infantile neurologic cutaneous articular syndrome (CINCA) describes the severe phenotype. The CAPS phenotypes display unspecific and unique clinical signs. Dermatologic, musculoskeletal, ocular, otologic, and neurologic disease symptoms combined with chronic systemic inflammation are characteristic. Nevertheless, making the CAPS diagnosis is challenging as several patients show a heterogeneous multi-system clinical presentation and the spectrum of genetic variants is growing. Somatic mosaicisms and low-penetrance variants lead to atypical clinical symptoms and disease courses. To avoid morbidity and to reduce mortality, early diagnosis is crucial, and a targeted anti-IL-1 therapy should be started as soon as possible. Furthermore, continuous and precise monitoring of disease activity, organ damage, and health-related quality of life is important. This review summarizes the current evidence in diagnosis and management of patients with CAPS.

## 1. Introduction

Autoinflammatory diseases (AID) are rare, often severe illnesses caused by genetic variants in innate immunity genes resulting in a constitutive overproduction of proinflammatory cytokines [1,2]. The genetic origin of monogenic interleukin-1 (IL-1) mediated AID was first determined for the familial Mediterranean fever (FMF) in 1997 [3,4]. In 1999, mutations in the *TNFRSF1A* gene were shown to be associated with Hibernian fever subsequently relabeled as tumor necrosis factor (TNF) receptor-associated periodic syndrome (TRAPS) [5,6]. Furthermore, for the hyperimmunoglobulinemia D syndrome (HIDS)/mevalonate kinase deficiency (MKD) the *MVK* gene was described in 1999 [7,8]. In 2001/2002, the *NLRP3* gene (also known as *CIAS1* or *NALP3* gene) was discovered, coding for the protein cryopyrin or synonymously called NLRP3/NALP3 protein [9,10,11]. Variants of this gene usually cause the cryopyrin-associated periodic syndromes (CAPS), a clinical spectrum of different autoinflammatory phenotypes with varying disease activity and phenotype-related risk for morbidity and mortality [12]. The CAPS spectrum includes mild, moderate, and severe phenotypes. The mild phenotype is also called familial cold autoinflammatory syndrome (FCAS, OMIM 120100), the moderate phenotype is known as Muckle–Wells syndrome (MWS, OMIM 191900), and the neonatal-onset multisystem inflammatory disease (NOMID)/chronic infantile neurologic cutaneous articular syndrome (CINCA) (OMIM 607115) describes the severe phenotype. In a recent consensus proposal of a new taxonomy for monogenetic AID, it was proposed to use the name *NLRP3*-associated autoinflammatory diseases (*NLRP3*-AID) for the CAPS spectrum [13]. The different levels of phenotypic severity of the same disease should be reflected by using the adjectives: mild, moderate, and severe [13]. However, it seems that this new taxonomy has yet failed to receive broad recognition, therefore, the, up until now, more prevalent term CAPS is used in this paper.

The CAPS phenotypes display unspecific and unique clinical signs. Dermatologic, musculoskeletal, ocular, otologic, and neurologic disease symptoms combined with chronic systemic inflammation are characteristic. Nevertheless, making the CAPS diagnosis is challenging as several patients show a heterogeneous multi-system clinical presentation and the spectrum of genetic variants is growing. Somatic mosaicisms and frequent variants of uncertain significance also known as low-penetrance variants lead to atypical clinical symptoms and disease courses.

The CAPS phenotypes are an important differential diagnosis in patients with systemic inflammation and suspected AID. Prompt diagnosis and early start of targeted anti-IL-1 treatment is crucial to avoid disease burden and organ damage. Furthermore, effective multidisciplinary management of patients with CAPS including, treat-to-target (T2T) strategies, as well as standardized monitoring of disease activity, organ damage, and disease-related psychosocial burden is important. In this review, we summarize what we know today, nearly 20 years after *NLRP3* gene discovery, give an overview of the current evidence in making the diagnosis, and give an update regarding the current management recommendations for patients with CAPS.

## 2. Epidemiology

CAPS belong to the orphan diseases or so-called rare diseases. Their true incidence is unknown due to underdiagnosis, underreporting, and selection bias, similar to other rare disease [14]. However, the prevalence is estimated to be 2.7 to 5.5 per 1 million and might be higher, as CAPS is still not widely known, and therefore often not diagnosed correctly [15,16]. The different CAPS phenotypes seem to vary in incidence and prevalence over the globe. Caucasians are more often affected, whereas no gender differences could be observed so far [15,17]. CAPS has been reported on nearly every continent, and the geographical distribution of CAPS might be influenced by external factors such as weather [14]. For example, patients with FCAS can avoid flares if not exposed to cold, and therefore they might prefer to live in areas with a mild climate. In North America, a founder mutation (L353P) associated with the mild CAPS phenotype FCAS is observed in up to 75% of CAPS patients, whereas in Europe the moderate MWS seems to be the most common CAPS phenotype [14,18,19]. The more severe phenotypes, such as CINCA or NOMID, are rare and mostly caused by de novo variants [20].

## 3. Genetics

In 2001, heterozygous gain-of-function variants in the *NLRP3* gene were identified in patients with FCAS and MWS and later in NOMID/CINCA [9,10,11]. This discovery led to the conclusion that FCAS, MWS, and NOMID/CINCA represent different phenotypes that belong to the same disease spectrum, called CAPS [12]. Today, genetic variants can be classified as ”pathogenic”, ”likely pathogenic”, ”uncertain significance”, ”likely benign”, and ”benign” [21]. The Infevers database (https://infevers.umai-montpellier.fr/web/), an exhaustive registry for sequence variants identified in different AID related genes, listed more than 240 sequence variants of the *NLRP3* gene in November 2020 [22]. Of these, more than 100 are known to be pathogenic/likely pathogenic and the majority is located in exon 3.

### 3.1. Frequent Variants of Uncertain Significance

Frequent variants of uncertain significance (VUS), also known as low-penetrance variants, can be present in asymptomatic healthy individuals. Nevertheless, some of these frequent VUS, also described as risk alleles, may contribute to an AID phenotype in affected carriers [23,24,25]. The systemic inflammation might be mediated by different pathways parallel to the caspase 1 activation, including IL-1β and non-IL-1β mediated inflammatory pathways [23,26]. Schuh et al. analyzed peripheral blood mononuclear cells of several symptomatic patients with *NLRP3* VUS and found increased NLRP3-specific IL-1β release upon stimulation and elevated NLRP3-independent IL-6 and TNF-α levels [27]. Furthermore, frequent VUS seem to act as susceptibility alleles to inflammation [28,29]. Well known frequent VUS in the *NLRP3* gene are the following variants: V198M, R488K, and Q703K. Symptomatic carriers display a distinct clinical phenotype, which includes typical CAPS symptoms of headache, urticarial-like rashes, and arthralgia, as well as atypical CAPS symptoms, such as severe gastrointestinal symptoms [23]. In addition, symptomatic patients with *NLRP3* VUS seem to have significantly more fever (76%) [23] and can present with cranial nerve inflammation [27]. Moreover, it seems that Q703K variants can be also associated with pharyngitis and oral aphthosis [24]. Whereas Kuemmerle-Deschner et al. stated that patients with frequent VUS in the *NLRP3* gene were at lower risk for eye disease, hearing loss, and renal involvement [23], Theodoropoulou et al. concluded that patients with clinical CAPS phenotype and Q703K variants had a comparable complication risk to patients with pathogenic *NLRP3* gene variants [24]. However, patients with low-penetrance *NLRP3* gene variants seem to display an intermediate biologic phenotype, with traditional markers of inflammation being elevated less frequently [23]. It is important to notice that the detection of a frequent VUS in the *NLRP3* gene does not genetically confirm the diagnosis of CAPS.

### 3.2. Somatic Mutation/Somatic Mosaicism

Somatic mutation/somatic mosaicism is a term which describes the occurrence of a new mutation post-zygotically in an embryo after the single cell stage with inheritance by all subsequent cells of that lineage, resulting in genetically different cell populations within an individual [30]. Whereas germline mutations are present in the first fertilized egg and, consequently, expressed in all cells of the body, the body distribution of somatic mutations depends on the time when the post-zygotic mutation occurs. If the somatic mutation occurs early in embryonic development, it results in a high frequency of altered cells across many different tissues and cell types; while those occurring later affect a lower frequency of mutant cells in a more limited distribution, potentially leading to a delayed onset of disease [30]. In 2005, Saito et al. identified a somatic mutation in a CINCA/NOMID patient [31]. Subsequently, somatic mosaicism was reported in 70% of former genetically negative NOMID/CINCA patients [32]. Labrousse et al. estimated that the proportion of CAPS-like patients carrying mosaicism ranged between 0.5% and 19% [33]. One of the most common somatic mutations is the E567K [33]. Up to now, there are 35 different somatic mutations that have been identified in the *NLRP3* gene [33]. Somatic mutations can result in an atypical AID phenotype, milder disease course, or late onset [32,34,35,36]. Furthermore, vertical transmission of somatic mosaicism has been reported [37]. Additionally, the phenotypic spectrum of CAPS appears to be related to the germinal/mosaic status and localization of the underlying variant [38]. Louvrier et al. reported that somatic mutations for *NLRP3* were mainly situated in the core of the NLRP3-inflammasome activating domain, while germline mutations were scattered throughout this domain [38]. Furthermore, it seems that there are two hotspots for somatic mutations. One is located in the HD2 domain of *NLRP3* and the second mosaic mutational hotspot involves Phe304 to Gly309 amino acids that overlap the Walker B motif of the nucleotide binding domain [38]. Due to the low or extremely low frequency of the mutant allele, somatic mutations can be missed using conventional methods of genetic analysis, such as Sanger sequencing. To detect somatic mutations, usually novel technologies are needed, such as next generation sequencing (NGS)-based methods with greater depth.

## 4. Pathogenesis

The *NLRP3* gene encodes for the protein NLRP3, which is part of the cytoplasmatic nucleotid-binding domain, a family member of the intracellular “NOD like” receptor (NLR) [39]. NLRP3 nucleates an intracellular multi-molecular complex, called the NLRP3 inflammasome [40]. The NLRP3 inflammasome consists of specific adaptor proteins such as ASC (apoptosis-associated speck-like protein containing a caspase recruitment domain) and several chaperone proteins [41,42] and the formation of this complex enables the activation of proinflammatory protease caspase-1. Caspase-1 can cleave pro-interleukin (IL)-1β and pro-IL-18 in their biological active forms (IL-1β, IL-18) [42,43,44]. IL-1β, and to a less extent IL-18, can elicit neutrophilic inflammation [14]. Once released, IL-1β causes a cascade of downstream signals, which finally result in the activation of nuclear factor κB (NFκB) and the production and release of other inflammatory cytokines. The NLRP3 inflammasome can be activated by a large variety of pathogen-associated molecular patterns (PAMPs) and danger-associated molecular patterns (DAMPs). Additionally, it seems that cells of CAPS patients have increased levels of reactive oxygen species due to increased redox stress, resulting in overactivation or ineffective anti-inflammatory mechanisms [45]. A unique feature of monocytes isolated from patients with FCAS is inflammasome activation when cultured at a slightly cooler temperature of 32 °C instead of the traditional 37 °C, resulting in increased IL-1β, IL-6, und TNF-α secretion [46].

## 5. Three Distinct Phenotypes Versus One Cryopyrin-Associated Periodic Syndromes (CAPS) Spectrum

Historically, FCAS, MWS, and NOMID/CINCA have been described as three distinct diseases. The first clinical reports of FCAS date back to 1940, when Kile and Rusk described FCAS as an inherited disorder with cold-induced skin and musculoskeletal symptoms [47]. MWS was first described in 1962 by Muckle and Wells as a syndrome of urticarial rash, neurosensory hearing loss, and amyloidosis [48]. CINCA/NOMID was first described by Prieur, in 1980, as a chronic inflammatory disease with rash, articular involvement, and chronic aseptic meningitis [49,50]. However, in all three phenotypes, the disease-causing variant was identified in the *NLRP3* gene [12]. Furthermore, patients can present with overlapping symptoms between the historically distinct phenotypes. Therefore, today CAPS is conceived as a continuous spectrum of disease. Although anti-IL-1 treatment is recommended for all phenotypes and is known to be effective throughout the complete CAPS severity spectrum [51], it is still important to distinguish among the subphenotypes, particularly in the moderate to severe CAPS phenotypes, because more intensive treatment is necessary to achieve remission and to prevent organ damage [52,53,54].

## 6. Clinical Manifestations

Similar to several other AID, CAPS is a multi-system inflammatory disease, affecting eyes, skin, muscles, joints, bones, kidneys, and the central nervous system. Some signs of inflammation are commonly associated with distinct subtypes of the CAPS spectrum (Table 1), whereas others are present in all subgroups. Characteristic CAPS symptoms can result from acute inflammation (flares) but they can also be caused by organ damage due to chronic inflammation. A chronic disease course was reported by 57% of 136 patients with CAPS, whereas 43% experienced only symptoms during acute inflammatory flares [55]. The age of CAPS onset ranges between perinatal/early infancy and adulthood. The median disease onset is 0.8 years (0.1–5), but a late-onset, with a median age of 50 years in patients with somatic mutations, has been described [36,55]. The duration of acute inflammatory flares can vary between <24 h up to more than 3 days [55]. CAPS flares can be triggered typically by cold, stress, infections, or trauma and lack of sleep [55]. In particular, cold is a commonly reported and potent trigger for the mild CAPS phenotypes, such as FCAS. In FCAS, inflammatory flares might be more frequent in the winter, on damp and windy days, and following exposure to air conditioning [14,56].

### 6.1. Unspecific General Symptoms

Common unspecific signs associated with CAPS are fever/subfebrile temperature, fatigue, and influenza-like muscle pains. While CAPS is classified as a hereditary fever disorder, it is important to know that fever is not always a complaint and, often, objective measurement of body temperature in patients with CAPS does not meet standard criteria for fever [14]. In particular, fatigue is a major component of CAPS and, together with emotional irritability, both can affect a patient’s quality of life [57,58].

### 6.2. Skin Manifestation

The characteristic dermatological manifestation of CAPS is a neutrophilic dermatitis that presents clinically with “urticaria-like” lesions, but it can appear also as erythematous and edematous papules or plaques. The rashes are rarely itchy, but often painful and sensitive to touch [14]. Typically, the rashes are located at the trunk and limbs, but can be seen also on the face, upper arms, thighs, and abdomen [14]. In the mild CAPS phenotypes, such as FCAS, the rashes are usually not induced by direct contact with cold objects or water, but often appear 1–4 h after cold exposure in areas not necessarily subjected directly to cold. Additionally, painful extremity swelling is reported [59]. Histologically perivascular neutrophilic infiltrations with leucozytoclasia without vasculitis and eosinophilic infiltrations can be detected in a skin biopsy [60,61].

### 6.3. Musculoskeletal Involvement

The involvement of muscles, bones, and joints depends on the clinical phenotype. Whereas patients with a mild CAPS phenotype may complain about limb pain, painful periarticular swelling and myalgia limited to inflammatory flares, patients with moderate CAPS often also experience arthralgia and arthritis [58,59]. Joints such as wrists, knees, and ankles are often affected [58]. Patients with severe CAPS may have skeletal abnormalities with bone deformation and may suffer from chronic polyarthritis. Several patients with NOMID/CINCA show characteristic arthropathy with bone and joint deformation caused by overgrowth and asymmetry of the cartilage, excessive uncontrolled growth of the patella and of the long bones, and abnormal epiphyseal and metaphyseal calcification [56,62]. Osseous lesions often affect growth plates asymmetrically with unilaterally reduced longitudinal growth of affected bones causing severe asymmetric limb length discrepancies [14]. In one third of patients, the arthropathy and bone changes are disabling [56]. Other features in patients with severe CAPS are chronic hydrocephalus, atypical facies with frontal bossing, macrocrania, and flattening of the nasal dorsum (“saddle nose”) [56,63,64].

### 6.4. Eye Involvement

Interstitial keratitis, conjunctivitis, episcleritis, iridocyclitis and anterior and posterior uveitis, band keratopathy, and corneal abnormalities can be present in patients with CAPS [65,66]. Less common are posterior stromal corneal opacification with edema, anterior iris snychecia, and cataract [65]. The most common eye manifestation is the conjunctivitis occurring during flares in many CAPS patients [65]. Patients with moderate to severe CAPS often report dry eyes with chronic conjunctivitis or perilimbal redness. In up to 40% of patients, the cornea is involved [58]. Chronic anterior uveitis and anterior segment manifestation varying from mild to severe are seen in up to 55% of patients with NOMID/CINCA [67]. Inflammation of the posterior eye segments is less frequent and can be present as vitritis, retinal vasculitis, and focal chorioretinitis. Elevated intracranial pressure in patients with severe CAPS (NOMID/CINCA) may cause papillary edema and subsequent optic disc atrophy [67]. Typically, ocular manifestations present bilaterally [68]. In more than 80% of NOMID/CINCA patients, the optic nerve head is affected, the most frequent ocular manifestation in this group of patients [58]. Ocular manifestations can progress to blindness and ocular disability.

### 6.5. Hearing Loss

Neurosensory hearing loss is a major symptom in moderate and severe CAPS. Usually, in untreated CAPS patients, hearing loss starts in childhood and early adulthood [56]. At onset, initially high frequencies are affected, which are often not detected in the routine otologic assessment [69,70]. Therefore, regular monitoring to provide early detection of hearing loss with high frequency pure tone averages (HF-PTA) is important [71]. In some patients, it is possible to detect a cochlear enhancement in the FLAIR magnet resonance imaging, representing inflammation of the inner ear [69]. The mechanism of hearing loss in CAPS is still under research. Nakanashi et al. raised the hypothesis that macrophages/monocyte-like cells in the cochlea might mediate local autoinflammation via activation the NLRP3 inflammasome [72]. They demonstrated that the inflammasome could be activated in macrophage/monocyte-like cells in a mouse cochlea with secretion of IL-1β and concluded that local cochlear activation of the NLRP3 inflammasome could induce cochlear autoinflammation and sensorineural hearing loss [72]. Depending on the type of variant, the hearing loss increases in extent and intensity throughout the course of the disease and with age [73]. Particularly, the variants T348M and E311K are associated with progressive linear deafness if patients are untreated [73], whereas the variant R918Q seems to cause a late onset of hearing loss and moderate progression [74]. A reversal or halt in progress of hearing loss may be achieved by timely induction of treatment but it can be irreversible if the start of treatment is delayed [75,76].

### 6.6. Central Nervous Impairment

Abnormalities of the central nervous system (CNS) can be caused by aseptic meningitis, in which polymorphonuclear cells infiltrate the cerebrospinal fluid (CSF) [56]. The CNS involvement varies with CAPS phenotype. In moderate CAPS, aseptic meningitis may occur only during inflammatory flares with headache and vomiting, whereas chronic aseptic meningitis and increased intracranial pressure including its consequences, such as chronic headache, papilledema, and CNS degeneration, is frequently observed in severe CAPS [56]. Brain atrophy and cognitive impairment may occur, depending on the severity of the disease. Mild cognitive deficits with need for specialized educational support are reported for the mild to moderate CAPS phenotypes [77]. Further CNS symptoms are seizures, strokes, and stroke-like episodes with hemiparesis, and vascular occlusions [64,78] have been reported. Early onset of CAPS is predictive of more severe CNS involvement and neurological complications [55].

## 7. Diagnostic Approach

**Figure 1:**
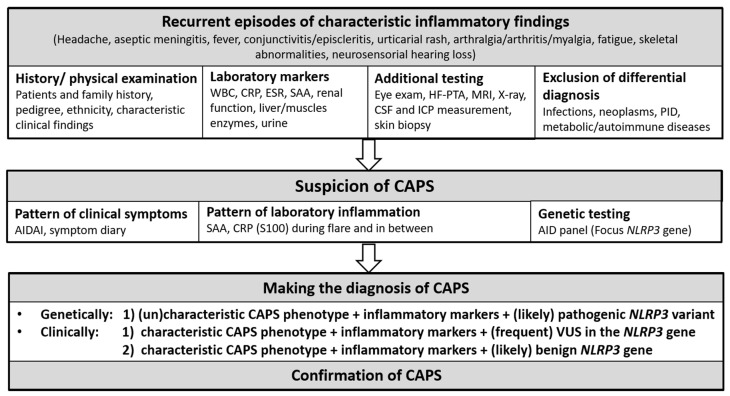
Diagnostic approach to CAPS. WBC, whole blood count; CRP, c reactive protein; ESR, erythrocyte sedimentation rate; SAA, serum amyloid A; HF-PTA, high frequency pure tone audiogram; MRI, magnet resonance imaging; CSF, cerebrospinal fluid; ICP, intracranial pressure; PID, primary immune deficiency; AIDAI, autoinflammatory disease activity index; S100, S 100 proteins (S100A12, S100A8/A9); VUS, variant of uncertain significance. Bold format and grey background indicate headings.

A median delay between symptom onset and CAPS diagnosis has been reported to be 1.4 years (0.2–8.9) [79]. Particularly, in the mild phenotypes, a diagnosis is often delayed (median age 23.3 years) as compared with the more severe CAPS phenotypes [80]. Although early age of onset is a very strong indicator for CAPS, diagnosis of CAPS also has to be considered in adults due to the rarity of the disease, mild phenotypes, and somatic mutation. If CAPS is suggested, a systematic stepwise diagnostic approach (Figure 1) similar to other AID is recommended including patient’s history, family history, physical examinations, and inflammatory markers during inflammatory flares and symptom-free intervals [81,82]. Red flags in patient history are specific triggers, such as cold exposure, characteristic disease symptoms, or a family history of early hearing loss or renal transplants. The autoinflammatory disease activity index (AIDAI), a standardized symptom diary [83], captures AID characteristic symptoms and can help to identify CAPS phenotypic patterns. Furthermore, a complete and thorough physical examination is important. The patients should be examined for typical clinical CAPS manifestations, such as urticarial-like rashes. In addition, laboratory inflammatory markers, such as the c-reactive protein (CRP), serum amyloid A (SAA), and the whole blood count, are considered to be first line laboratory examinations during inflammatory flares and in symptom-free intervals [82]. Characteristics of systemic inflammation are blood leukocytosis, neutrophilia, thrombocytosis, anemia, increased erythrocyte sedimentation rate (ESR), elevated CRP and SAA, and myeloid-related protein 8 and 14 (MRP8/MRP14, also known as S100A8/S100A9) [84,85,86]. Particularly, SAA is one crucial parameter to detect subclinical inflammation and risk evaluation for the development of AA-amyloidosis [87]. Additionally, S100A12 and MRP8/MRP14 can be used for the monitoring of inflammation with a good correlation to inflammation and treatment response [86,88]. Other disorders associated with recurrent systemic inflammation, such as immunodeficiencies, infections, autoimmune diseases, and malignancies, need to be excluded. If these first steps support the suspicion of CAPS, musculoskeletal, neurological, and ophthalmologic examination is suggested [51]. Moreover, HF-PTA, including 0.5 to 10 kHz, formal cognitive testing, brain MRI studies, lumbar punctures with opening pressure, cell counts, protein concentration, and lesional skin biopsy should be considered [51]. During inflammatory flares, elevated neopterin and elevated protein can be detected in the CSF [89]. In patients with severe musculoskeletal involvement, X-ray and bone MRI should be performed [51]. Molecular diagnosis should be attempted when the clinical phenotype, laboratory, and functional tests are suggestive for CAPS.

### 7.1. Diagnostic and Classification Criteria

#### 7.1.1. Diagnostic Criteria

CAPS is diagnosed clinically and genetically. Diagnostic criteria are used to guide the care of individual patients, and therefore must have a very high sensitivity and specificity in order that patients receive the correct diagnosis and treatment [90]. The diagnostic criteria of CAPS recognize that all but a few patients with CAPS have detectable systemic inflammation and use unique CAPS-specific clinical features along the whole disease spectrum to achieve reasonable specificity and sensitivity to aid clinicians in making the CAPS diagnosis [91]. These diagnostic criteria do not include genetic confirmation, and therefore can be applied in places where genetic testing is not available. If genetic testing is not available or it is negative, making a CAPS diagnosis is possible if raised inflammatory markers (CRP/SAA) can be detected plus at least two of the following symptoms: urticarial-like rash (neutrophilic dermatitis), cold-triggered episodes, sensorineural hearing loss, musculoskeletal symptoms, chronic aseptic meningitis, and skeletal abnormalities (Table 2) [91].

#### 7.1.2. Classification Criteria

Classification criteria are primarily used to define cohorts of patients that can be included in clinical research. Using classification criteria may result in some patients with the disease not being captured (false negative); however, the chance of patients not having the indicated diagnosis (false positive) is very low [90]. In 2019, Gattorno et al. developed validated evidence-based classification criteria for hereditary AID with high sensitivity and specificity [92]. The classification criteria for CAPS are summarized in Table 3.

### 7.2. Diagnostic Challenges

As mentioned above, patients with low-penetrance variants or somatic mosaicism might present with atypical clinical CAPS phenotypes. AID panels and targeted NGS may be negative or inconclusive and the correlation of clinical phenotype and genetic result is critical [93]. Furthermore, patients might present with a heterogeneous multi-systemic clinical presentation. Advanced genetic testing can enable a diagnosis in some AID patients [94,95].

## 8. Treatment

CAPS treatment is a multidisciplinary effort including medication, psychosocial support, physiotherapy and supportive care. Treatment aims are to suppress systemic inflammation, to improve functionality, to prevent organ damage, and to increase patients’ quality of life. To achieve these aims, cytokine targeting drugs are important and evidence-based treatment plans including treat-to-target (T2T) strategies play a pivotal role in CAPS management [51,96]. The key component of T2T is the definition of a clinical target, such as disease remission or the lowest possible disease activity. Standardized and repeated examinations are required to determine if a previously defined target is achieved [97]. Different levels of disease activity may require different treatment selections and dosing approaches [51,96]. Since IL-1 plays a central role in CAPS pathogenesis, the anti-IL-1 treatment is recommended for the whole CAPS spectrum [51]. Currently, three anti-IL-1 treatments (anakinra, canakinumab, and rilonacept) are available, and several studies have addressed their safety and efficacy. However, symptomatic patients with low-penetrance variants are at risk to achieve only a partial response to anti-IL-1 treatment, as inflammation seems to be mediated due to NLRP3 specific IL-1β release and NLRP3-independent IL-6 or TNF-α production [23,27].

### 8.1. Anti-IL-1 Treatment

Anakinra is a short-acting recombinant IL-1 receptor antagonist, which has been proven to have long-term efficacy and safety in several studies [54,98,99,100,101]. Anakinra is administered daily subcutaneously and blocks the binding of IL-1α and IL-1β to the IL-1 receptor. In a study of 43 CAPS patients treated with anakinra, for up to 5 years, the most reported serious adverse events were pneumonia and gastroenteritis [101]. Anakinra has been approved by the European Medicines Agency (EMA) and the Food and Drug Administration (FDA) for CAPS. For anakinra, the typical dosing regimen varies from 1 to 2 mg/kg/day for patients with FCAS, up to 10 mg/kg/day for critically ill patients with NOMID/CINCA [14]. The CNS penetrance of anakinra seems to be superior, and therefore this might be the treatment of choice in cases with aseptic meningitis [102]. The recombinant soluble IL-1 receptor rilonacept binds to IL-1α and IL-1β. Weekly subcutaneous administration has shown a good safety and efficacy profile against CAPS [103,104]. So far, rilonacept has only been approved by the FDA. The dose of rilonacept for adults is 160 mg/week and varies from 2.2 to 4.4 mg/kg/week in children [14]. Canakinumab is a fully humanized anti-IL-1β monoclonal antibody that selectively binds to soluble IL-1β and has to be administered subcutaneously every four to eight weeks. Several studies have confirmed long-term efficacy and safety against CAPS [52,105,106,107,108,109]. In patients with mild to moderate CAPS, 150 mg of canakinumab can be administered if the body weight is >40 kg or it can be dosed with 2 mg/kg for patients from ≥15 to ≤ 40 kg, every four to eight weeks [14]. For children (15–40 kg) with an inadequate response, a dose increase, up to 3–4 mg/kg, might be necessary and dosing up to 8 mg/kg every four weeks has been described for NOMID/CINCA patients [110]. Canakinumab has been approved by the EMA and FDA.

### 8.2. Supportive Therapy

In patients with CAPS, supportive care plays an important role and can consist, for example, of hearing aids, physiotherapy, and orthopedic devices. Furthermore, adjunctive therapies, such as non-steroidal anti-inflammatory drugs for pain and fever or corticosteroid eye drops and tear substitutes, might help to overcome the disease symptoms. Particularly for patients with the mild CAPS phenotype, warming therapies and local protection for cold (gloves, wristlets) can be beneficial for flair prophylaxis.

### 8.3. Psychosocial Needs

In addition to anti-IL-1 treatment, CAPS patients can profit from psychosocial support. AID have been shown to be associated with depression, lower health-related quality of life, anxiety, and risk of isolation due to frequent canceling of social events [111,112,113]. Since AID can affect all areas of life and well-being is linked to psychological factors such as illness beliefs, coping strategies, and the distribution of dependency, these aspects have to be taken into account in the long-term management of CAPS [111,114]. Furthermore, patient support networks can provide emotional support.

### 8.4. Outlook Drug Development

Currently, there are new treatment approaches under development, which might be used to treat CAPS in the future. For example, small molecule inhibitors targeting NLRP3 directly are one promising drug development [115]. The diarylsulfonylurea compound MCC950 seems to be a potent selective small inhibitor of NLRP3, blocking canonical and non-canonical NLRP3 activation by closing the “open” confirmation of active NLRP3 [116,117]. MCC950-based therapies may effectively treat inflammation driven by wild-type NLRP3, and an evaluation of its ability to inhibit CAPS mutant variants has provided a mechanistic framework for advancing therapeutic development and for understanding its therapeutic potential in patients [118]. Furthermore, Youm et al. showed that β-hydroxybutyrate (BHB) suppressed the activation of the NLRP3 inflammasome by preventing K+ efflux and reducing ASC oligomerization and speck formation [119]. In addition, several other ways to inhibit the NLRP3 inflammasome, such as autophagy or microRNAs, are under research [115].

## 9. Monitoring

Regular monitoring of disease activity is crucial to determine disease activity and organ damage [51]. Monitoring includes serial physical examinations, measurements of weight and height, audiology and ophthalmologic exams, radiographs and MRIs, as well as musculoskeletal, neurological, and laboratory examinations, such as blood count, liver and muscle enzymes, renal function, urine analysis, CSF measurements, and determination of SAA and CRP levels to detect ongoing inflammation. For monitoring of disease activity in CAPS, longitudinal patient diaries, such as the MWS disease activity score (MWS-DAS) or the AIDAI, can be used for systematic assessment of daily diseases symptoms. Both were initially developed for clinical trials but can be used by clinicians as well. The MWS-DAS captures disease symptoms in 10 domains; nine domains reflect the organ involvement in MWS (fever, headache, eye involvement, hearing impairment, oral ulcers, abdominal pain, renal disease, musculoskeletal disease, and rash), and the tenth is the patient’s global assessment score [100,120]. The validated AIDAI is a simple tool for outpatients to assess CAPS disease activity at home [83], allowing treating physicians to better differentiate between inactive or active disease and the need for treatment adjustments. The autoinflammatory disease damage index (ADDI) is a reliable instrument for assessing disease-related organ damage [121]. The ADDI consists of 18 items grouped into the following eight categories: reproductive, renal/amyloidosis, developmental, serosal, neurological, auditory, ocular, and musculoskeletal damage [121,122]. The ADDI can be used to monitor structural damage in individual patients and allows outcome analysis and comparison of results in clinical trials [121].

## 10. Prognosis

The prognosis in CAPS patients depends, on the one hand, on the CAPS phenotype and, on the other hand, on an early diagnosis allowing the start of effective treatment. The prevalence for AA-amyloidosis in CAPS patients without treatment varies between 10% for mild phenotypes and 25% for moderate phenotype [123]. With the availability of anti-IL-1 treatment, the prognosis of patients with CAPS has improved considerably. However, early and aggressive treatment is crucial to improve quality of life and to avoid organ damage. Only an early start of treatment will prevent organ damage and avoid progress. Furthermore, starting treatment early can result in reversibility, for example, of hearing loss.

## Figures and Tables

**Table 1:** Clinical manifestations and characteristics of cryopyrin-associated periodic syndromes (CAPS) (adapted from [14,18]).

	Clinical Manifestations and Characteristics of CAPS		
	Mild Phenotype (FCAS)	Moderate Phenotype (MWS)	Sever Phenotype (CINCA/NOMID)
**Disease onset**	<6 months–adulthood	Early childhood–adulthood	Perinatal
**Family history**	Often positive	Often positive	Often negative (sporadic de novo mutations)
**Inflammatory flares**	Yes	Yes + continuous disease symptoms	Yes + continuous disease symptoms
**Duration of inflammatory flares**	30 min–72 h	1–3 Days ± subclinical	Persistent inflammation
**Cold trigger**	Yes	Possible	Rare
**Dermatological manifestations**	Cold-induced neutrophilic urticaria	Neutrophilic urticaria	Neutrophilic urticaria
**Fever**	6–24 h after cold exposure possible	Particularly in childhood	Yes
**Fatigue**	Rare	Yes	Yes
**Hearing loss**	No	Yes	Yes
**Ocular manifestation**	Conjunctivitis	Conjunctivitis, episcleritis, optic disc edema/papilledema	Conjunctivitis, episcleritis, optic disc edema/papilledema
**Muskulosceletal manifestations**	Myalgia, arthralgia	Myalgia, arthralgia, oligoarthritis	Myalgia, arthralgia, (poly-) arthritis. epiphyseal bony overgrowth, limb-length discrepancies, contractures
**Central nervous system manifestations**	Headache	Headache, intermittent aseptic meningitis	Headache, chronic aseptic meningitis increased intracranial pressure, brain atrophy

Abbreviations: FCAS, familial cold autoinflammatory syndrome; MWS, Muckle–Wells syndrome; NOMID, neonatal-onset multisystem inflammatory disease; CINCA, chronic infantile neurologic cutaneous articular syndrome.

**Table 2:** Diagnostic criteria for CAPS (data from [91]).

Diagnostic Criteria for CAPS	
mandatory	+ ≥2 of 6 clinical characteristic symptoms/signs
Raised inflammatory markers (C-reactive protein, serum amyloid A)	Urticarial rash Cold/stress-triggered flares Chronic aseptic meningitis Neurosensorial hearing loss Muskoloskeletal symptoms (arthralgia, arthritis, myalgia) Skeletal abnormalities (epipysial overgrowth/frontal bossing)

**Table 3:** Classification criteria for CAPS (data from [92]).

Eurofever/Printo Classification Criteria for CAPS	
Genetic Criteria	Clinical Criteria
Presence of a pathogenic/likely pathogenic *NLRP3* gene variant	At least one among the following: Urticarial rash Red eye (conjunctivitis, episclereitis, uveietis) Neurosensorial hearing loss
Presence of a frequent *NLRP3* gene variant of uncertain significance	At least two among the following: Urticarial rash Red eye (conjunctivitis, episclereitis, uveitis) Neurosensorial hearing loss
No presence of one of the above mentioned *NLRP3* gene variants	At least two among the following: Urticarial rash Cold/stress-triggered flares Chronic aseptic meningitis Neurosensorial hearing loss Skeletal abnormalities (epipysial overgrowth/frontal bossing)
